# Supplementary material for: Governing principles of hydration of mixed proton conducting Co-based double perovskites
Source: Nat Commun. 2026 Mar 23;17:4344. doi: 10.1038/s41467-026-70212-w (PMC13171892; doi:10.1038/s41467-026-70212-w)
Supplement: Supplementary file 1 — Supplementary Information [file 41467_2026_70212_MOESM1_ESM.pdf]

Supplementary Information

Governing principles of hydration of mixed proton conducting Co-based double perovskites

Ragnar Strandbakke [a,b *], Sebastian Lech Wachowski [c], Maria Balaguer [d], Lasse Vines [e], Thomas Neset Sky[e], Iga Szpunar [c,f], Patricia A. Carvalho [a], Aleksandra Mielewczyk-Gryń [c], Magnus H. Sørby [g], Maria Gazda [c], Jose M. Serra [d], Truls Norby [b **]

[a] Department of Sustainable Energy Technology, SINTEF Industry, NO-0314 Oslo, Norway

[b] Department of Chemistry, Centre for Materials Science and Nanotechnology, University of Oslo, Gaustadalléen 21, NO-0349 Oslo, Norway

[c] Institute of Nanotechnology and Materials Engineering, Faculty of Applied Physics and Mathematics, and Advanced Materials Centre, Gdańsk University of Technology, ul. G. Narutowicza 11/12, 80-233 Gdańsk, Poland

[d] Instituto de Tecnología Química (ITQ), Consejo Superior de Investigaciones Científicas-Universitat Politècnica de València, 46022, Valencia, Spain

[e] Department of Physics, Centre for Materials Science and Nanotechnology, University of Oslo, Gaustadalléen 21, NO-0349 Oslo, Norway

[f] Wallenberg Initiative Materials Science for Sustainability, Department of Chemistry and Chemical Engineering, Chalmers University of Technology, Gothenburg 41296, Sweden

[g] Department for Neutron Materials Characterization, Institute for Energy Technology, P.O. Box 40, NO-2027 Kjeller, Norway

[*] Corresponding author: ragnar.strandbakke@sintef.no

[**] Corresponding author: truls.norby@kjemi.uio.no

Supplementary Table

Supplementary Table 1: Abbreviation, formulas and hydration levels of all measured compositions calculated from total mass gain after 2 hrs in wet air at 300°C. Closed symbols (also used in Fig. 2b and Fig. S1) indicate compositions with closed-shell lanthanides, partly filled symbols indicate mainly closed-shell lanthanides, and open symbols indicate mainly or fully open-shell lanthanides. Closed symbols with colours are previously reported PCEC electrode materials or compositions of particular significance.

Label	Formula	Hydration / mol% H_2O	Mol% H+ from D_2O/H_2O	Symbol in fig. 1b	Space group	Lattice parameters	Rietveld fit quality	Sample and annealing info, reference, etc.
BGC	$BaGdCo_2O_{6-\delta}$	1.13	na	⬢	orthorhombic *Pmmm*	a = 3.87707(1) Å<br>b = 7.82650(2) Å<br>c = 7.53379(1) Å	Rwp(SR-PXD) = 11.85%<br>Rwp(PND) = 1.45%<br>Rwp(total) = 4.19%	Powder from crushed pellets, sintered at 1150 °C for 48h, combined PND and SR-PXD refinement, ref: https://doi.org/10.1016/j.actamat.2020.08.018
BGLC82	$BaGd_{0.8}La_{0.2}Co_2O_{6-\delta}$	0.82	0.4	■	orthorhombic *Pmmm* 78.8 *wt%* *Pbnm* $GdCoO_3$ 11.2 wt % *	a = 3.8842 Å<br>b = 7.8635<br>c = 7.58319 Å	Rwp = 12.8%<br>Gof = 1.5	As-prepared powders from Marion Technology combined PND and SR-PXD Refinement ref: https://doi.org/10.1039/D2DT02277J
BGLC82	$BaGd_{0.8}La_{0.2}Co_2O_{6-\delta}$	0.96	na	Not in figure 2b	tetragonal P4/mmm	a = 3.8961(1)<br>c = 7.5771(3)	Rwp = 27.9%<br>Chi^2 = 2.5	Powder from crushed pellets, sintered at 1150 °C for 48h, XRD collected in 2θ angle from 20° to 90° with a Phillips X'Pert Pro diffractometer with Cu Kα radiation, proportional counter, and Bragg-Brentano geometry, GSAS II software used for refinement.
BGLC82 2a3b3c					orthorhombic *Pmmm*	a = 7.5820(9)<br>b = 11.6515(9)<br>c = 11.6837(9)	Rwp = 17.0 %<br>Gof = 1.5	Powders from Marion Tech after hydration combined PND and SR-PXD Refinement ref: https://doi.org/10.1039/D2DT02277J
$BGLC37_{red}$	$BaGd_{0.3}La_{0.7}Co_2O_{6-\delta}$	Air: 5.7<br>N_2: 0.04	Air: 3<br>N_2:1.24 (hydrogenation)	Not in figure 2b	tetragonal *P4/mmm*	a = 3.90710 Å<br>c = 7.65050 Å	Rwp = 2.1%<br>Gof = 3.8	Powder from crushed pellets, sintered at 1000 °C for 48h in Ar and 24 h in O_2 at 350 °C, SR-PXD Diamond (UK)
BGLC37	$BaGd_{0.3}La_{0.7}Co_2O_{6-\delta}$	0.78	0.9	◆	orthorhombic *Pmmm*	a = 3.88556 Å<br>b = 7.803 Å<br>c = 7.68708 Å	Rwp = 10.0%<br>Gof = 2.2	As-prepared powders from Marion Technology Refined from SR-PXD data (Elettra, It) with parameters input from NPD (PowGen, Oak Ridge, US)
BGLC37 2a3b3c					orthorhombic *pmmm*	a = 7.8021(3)<br>b = 11.5303(3)	Rwp = 9.7%	SR-PXD (Elettra, (It), Powders from Marion Tech after hydration

						c = 11.6582(5)	Gof = 2.1	
BLC(O)	$BaLaCo_2O_{6-\delta}$	0.82		★	tetragonal $P4/mmm$ 35 wt %. orthorhombic $Pmmm$ 65 wt %	a = 3.87672(8) Å c = 7.7819(3) Å ------------------- a = 3.9155(2) Å b = 7.8142(5) Å c = 7.7247(3) Å	Rwp(SR-PXD) = 5.8% Rwp(PND) = 5.4% Rwp(total) = 5.6%	Powder from crushed pellets, sintered at 1150 °C for 48h and post-annealing in Ar at 1050 °C for 24h, combined PND and SR-PXD refinement, ref: https://doi.org/10.1016/j.actamat.2020.08.018
BLSrC	$BaLa_{0.75}Sr_{0.25}Co_2O_{6-\delta}$	0.66	**na**	+	cubic $Pm\bar{3}m$	a = 3.8910(3) Å	Rwp = 1.9 % Chi^2 = 4.9	Powder from crushed pellets, sintered at 1150 °C for 48h, XRD collected in 2θ angle from 10° to 135° with a Phillips X'Pert Pro diffractometer with Cu Kα radiation, Pixcel 1D detector, and Bragg-Brentano geometry, GSAS II software used for refinement. Minor secondary peaks of another $Pm\bar{3}m$ phase with slighlty larger unit cell (a = 3.9267(1) Å) were detected.
BGLYC721	$BaGd_{0.7}La_{0.2}Y_{0.1}Co_2O_{6-\delta}$	0.50	na	■	tetragonal $P4/mmm$	a = 3.9004(1) Å c = 7.5618(1) Å	Rwp(total) = 2.0% Chi^2 = 5.7	Powder from crushed pellets, sintered at 1150 °C for 48h, XRD collected in 2θ angle from 10° to 135° with a Phillips X'Pert Pro diffractometer with Cu Kα radiation, Pixcel 1D detector, and Bragg-Brentano geometry, GSAS II software used for refinement.
BGLC28	$BaGd_{0.2}La_{0.8}Co_2O_{6-\delta}$	0.49	na	◆	cubic $Pm\bar{3}m$	NPD (Isotope sample): a = 3.8898 Standard sample: a = 3.893(1) Å	NPD (isotope sample): Rwp = 4.1 Gof = 8.0 PXD (Standard sample: Chi^2 = 2.5 Rwp = 23.7 %	Powder from crushed pellets, sintered at 1150 °C for 48h, XRD collected in 2θ angle from 20° to 90° with a Phillips X'Pert Pro diffractometer with Cu Kα radiation, proportional counter, and Bragg-Brentano geometry, GSAS II software used for refinement. Minor secondary peaks of another $Pm\bar{3}m$ phase with slighlty smaller unit cell (a = 3.837(1) Å) were detected. NPD on ^{160}Gd isotope enriched sample: (PowGen, Oak Ridge, US)
BNSmC55	$BaNd_{0.5}Sm_{0.5}Co_2O_{6-\delta}$	0.45	na	□	orthorhombic $Pmmm$	a = 3.90359(3) Å b = 7.79336(5) Å c = 7.59833(5) Å	Rwp = 2.6% Chi^2 = 2.5	Powder from crushed pellets, sintered at 1100 °C for 48h, XRD collected in 2θ angle from 15° to 100° with a PANalytical CubiX fast diffractometer, using CuKα1 radiation (λ=1.5406 Å) and an X'Celerator detector in Bragg-Brentano geometry, GSAS II software used for refinement.
BSmLC82	$BaSm_{0.8}La_{0.2}Co_2O_{6-\delta}$	0.43	na	□	orthorhombic $Pmmm$	a = 3.90297 (7) Å b = 7.7948 (1) Å c = 7.6016 (1) Å	Rwp = 3.55% Chi^2 = 5.4	Powder from crushed pellets, sintered at 1100 °C for 48h, XRD collected in 2θ angle from 15° to 100° with a PANalytical CubiX fast diffractometer, using CuKα1 radiation (λ=1.5406 Å) and an

								X'Celerator detector in Bragg-Brentano geometry, GSAS II software used for refinement.
BGLuC82	$BaGd_{0.8}Lu_{0.2}Co_2O_{6-\delta}$	0.38	na	⬟	tetragonal $P4/mmm$	a = 3.8842(1) Å<br>c = 7.5240(1) Å	Rwp = 2.3%<br>Chi^2 = 7.4	Powder from crushed pellets, sintered at 1150 °C for 48h, XRD collected in 2θ angle from 10° to 135° with a Phillips X'Pert Pro diffractometer with Cu Kα radiation, Pixcel 1D detector, and Bragg-Brentano geometry, GSAS II software used for refinement.
BGNLC712	$BaGd_{0.7}Nd_{0.1}La_{0.2}Co_2O_{6-\delta}$	0.29	na	▷	tetragonal $P4/mmm$	a = 3.8987(1) Å<br>c = 7.5754(1) Å	Rwp = 1.8%<br>Chi^2 = 5.8	Powder from crushed pellets, sintered at 1150 °C for 48h, XRD collected in 2θ angle from 10° to 135° with a Phillips X'Pert Pro diffractometer with Cu Kα radiation, Pixcel 1D detector, and Bragg-Brentano geometry, GSAS II software used for refinement.
BLNC82	$BaLa_{0.8}Nd_{0.2}Co_2O_{6-\delta}$	0.25	na	⊡	cubic $Pm\bar{3}m$	a = 3.88658(2) Å	Rwp = 2.7%<br>Chi^2 = 1.4	Powder from crushed pellets, sintered at 1100 °C for 48h, XRD collected in 2θ angle from 15° to 100° with a PANalytical CubiX fast diffractometer, using CuKα1 radiation (λ=1.5406 Å) and an X'Celerator detector in Bragg-Brentano geometry, GSAS II software used for refinement.
BDC	$BaDyCo_2O_{6-\delta}$	0.23	na	○	orthorhombic $Pmmm$	a = 3.86090(5) Å<br>b = 7.80014(11) Å<br>c = 7.5020(1) Å	Rwp = 7.6%	Powder from crushed pellets, sintered at 1150 °C for 48h, SR-PXD data used for refinement, ref: https://doi.org/10.1016/j.actamat.2020.08.018
BGLCT82	$BaGd_{0.8}La_{0.2}Co_{1.8}Ti_{0.2}O_{6-\delta}$	0.21	na	⊞	tetragonal $P4/mmm$ 44 wt %.<br><br>cubic $Pm\bar{3}m$ 56 wt %	a = 3.8914(4) Å<br>c = 7.755(2) Å<br><br>a = 3.8950(4) Å	Rwp = 3.6%<br>Chi^2 = 0.9	Powder from crushed pellets, sintered in Ar at 1000 °C, 48h and air at 350 ºC, 6 h. XRD collected in 2θ angle from 5° to 90° with a PANalytical CubiX fast diffractometer, using CuKα1 radiation (λ=1.5406 Å) and an X'Celerator detector in Bragg-Brentano geometry, GSAS II software used for refinement
BGLYC622	$BaGd_{0.6}La_{0.2}Y_{0.2}Co_2O_{6-\delta}$	0.21	na	◆	tetragonal $P4/mmm$	a = 3.9040(1) Å<br>c = 7.5539(1) Å	Rwp = 1.3%<br>Chi^2 = 2.7	Powder from crushed pellets, sintered at 1150 °C for 48h, XRD collected in 2θ angle from 10° to 135° with a Phillips X'Pert Pro diffractometer with Cu Kα radiation Pixcel 1D detector, and Bragg-Brentano geometry, GSAS II software used for refinement.
BLPC55	$BaLa_{0.5}Pr_{0.5}Co_2O_{6-\delta}$	0.20	na	□	tetragonal $P4/mmm$ 48 wt %.<br><br>cubic $Pm\bar{3}m$ 54 wt %	a = 3.9037(6) Å<br>c = 7.6781(5) Å<br><br>a = 3.8835(2) Å	Rwp = 1.6%<br>Chi^2 = 5.6	Powder from crushed pellets, sintered at 1150 °C for 48h, XRD collected in 2θ angle from 10° to 135° with a Phillips X'Pert Pro diffractometer with Cu Kα radiation Pixcel 1D detector, and Bragg-Brentano geometry, GSAS II software used for refinement. .
BSrGLC28	$Ba_{0.5}Sr_{0.5}Gd_{0.2}La_{0.8}Co_2O_{6-\delta}$	0.19	na	⊖	cubic $Pm\bar{3}m$	a = 3.8539(5) Å	Rwp = 1.8%<br>Chi^2 = 7.6	Powder from crushed pellets, sintered at 1100 °C for 48h, XRD collected in 2θ angle from 10° to 135° with a Phillips X'Pert Pro

								diffractometer with Cu Kα radiation Pixcel 1D detector, and Bragg-Brentano geometry, GSAS II software used for refinement.
BSrGC	$Ba_{0.5}Sr_{0.5}GdCo_2O_{6-\delta}$	0.18	na	⊠	tetragonal<br>$P4/mmm$	a = 3.8510(3) Å<br>c = 7.5370(5) Å	Rwp = 1.3%<br>Chi² = 3.5	Powder from crushed pellets, sintered at 1100 °C for 48h, , XRD collected in 2θ angle from 10° to 135° with a Phillips X'Pert Pro diffractometer with Cu Kα radiation Pixcel 1D detector, and Bragg-Brentano geometry, GSAS II software used for refinement.
BGLSrC	$BaGd_{0.2}La_{0.55}Sr_{0.25}Co_2O_{6-\delta}$	0.15	na	⊟	cubic<br>$Pm\bar{3}m$	a = 3.8999(3) Å<br>c = 7.8067(2) Å	Rwp = 1.8%<br>Chi² = 7.2	Powder from crushed pellets, sintered at 1100 °C for 48h, , XRD collected in 2θ angle from 10° to 135° with a Phillips X'Pert Pro diffractometer with Cu Kα radiation Pixcel 1D detector, and Bragg-Brentano geometry, GSAS II software used for refinement
BSrGLC82	$Ba_{0.5}Sr_{0.5}Gd_{0.8}La_{0.2}Co_2O_{6-\delta}$	0.13	na	⦶	tetragonal<br>$P4/mmm$<br>78 wt %.<br><br>cubic<br>$Pm\bar{3}m$<br>22 wt %	a = 3.859(1) Å<br>c = 7.553(1) Å<br><br>a = 3.823(1) Å	Rwp = 17.8%<br>Chi² = 1.7	Powder from crushed pellets, sintered at 1100 °C for 48h, XRD collected in 2θ angle from 10° to 90° with a Phillips X'Pert Pro diffractometer with Cu Kα radiation, proportional counter, and Bragg-Brentano geometry, GSAS II software used for refinement.
BSrGLCT82	$Ba_{0.5}Sr_{0.5}Gd_{0.8}La_{0.2}Co_{1.8}Ti_{0.2}O_{6-\delta}$	0.12	na	×	tetragonal<br>$P4/mmm$<br>67.5 wt %.<br><br>cubic<br>$Pm\bar{3}m$<br>32.5 wt %	a = 3.8582(1) Å<br>c = 7.6077 (5) Å<br><br>a = 3.8403 (1) Å	Rwp = 3.3%<br>Chi² = 1.9	Powder from crushed pellets, sintered at 1100 °C for 48h, XRD collected in 2θ angle from 15° to 100° with a PANalytical CubiX fast diffractometer, using CuKα1 radiation (λ=1.5406 Å) and an X'Celerator detector in Bragg-Brentano geometry, GSAS II software used for refinement.
BLC(C)	$Ba_{0.5}La_{0.5}CoO_3$	0.10	na	★	cubic $Pm\bar{3}m$	a = 3.8850(6) Å	R_{wp}(SR-PXD) = 5.2%<br>R_{wp}(PND) = 4.2%<br>R_{wp}(total) = 4.7%	Powder from crushed pellets, sintered at 1150 °C for 48h, SR-PXD data used for refinement, ref: https://doi.org/10.1016/j.actamat.2020.08.018
BLPC28	$BaLa_{0.2}Pr_{0.8}Co_2O_{6-\delta}$	0.06	na	□	orthorhombic<br>$Pmmm$	a = 3.8997(1) Å<br>b = 7.8122(3) Å<br>c = 7.6546(2) Å	Rwp = 2.8%<br>Chi² = 1.8	Powder from crushed pellets, sintered at 1100 °C for 48h, XRD collected in 2θ angle from 15° to 100° with a PANalytical CubiX fast diffractometer, using CuKα1 radiation (λ=1.5406 Å) and an X'Celerator detector in Bragg-Brentano geometry, GSAS II software used for refinement. Cell code 230741-ICSD.
BSrPC	$Ba_{0.5}Sr_{0.5}PrCo_2O_{6-\delta}$	0.06	na	□	cubic<br>$Pm\bar{3}m$	a = 3.8432(1) Å	Rwp = 1.9%<br>Chi² = 6.3	Powder from crushed pellets, sintered at 1100 °C for 48h, XRD collected in 2θ angle from 10° to 135° with a Phillips X'Pert Pro diffractometer with Cu Kα radiation, Pixcel 1D detector, and Bragg-Brentano geometry, GSAS II software used for refinement

BSmLuC82	$BaSm_{0.8}Lu_{0.2}Co_2O_{6-\delta}$	0.05	na	□	tetragonal $P4/mmm$	a = 3.8908(1) Å<br>c = 7.5542(1) Å	Rwp = 1.8%<br>Chi^2 = 4.4	Powder from crushed pellets, sintered at 1150 °C for 48h, XRD collected in 2θ angle from 10° to 135° with a Phillips X'Pert Pro diffractometer with Cu Kα radiation, Pixcel 1D detector, and Bragg-Brentano geometry, GSAS II software used for refinement. Minor secondary phases peaks present.
BSmTC82	$BaSm_{0.8}Tb_{0.2}Co_2O_{6-\delta}$	0.04	na	□	orthorhombic $Pmmm$	a = 3.8941(3) Å<br>b = 7.8321(7) Å<br>c = 7.5473(6) Å	Rwp = 5.3%<br>Chi^2 = 1.0	Powder from crushed pellets, initially sintered at 1000 °C for 48h, A second calcination was performed at 1100 ºC, 12 h. XRD collected in 2θ angle from 15° to 100° with a PANalytical CubiX fast diffractometer, using CuKα1 radiation (λ=1.5406 Å) and an X'Celerator detector in Bragg-Brentano geometry, GSAS II software used for refinement.
BGLC55	$BaGd_{0.5}La_{0.5}Co_2O_{6-\delta}$	0.02	na	▲	tetragonal $P4/mmm$	a=3.8958 (1) Å<br>c=7.6285 (3) Å	Rwp = 5.1%<br>Chi^2 = 1.9	Powder from crushed pellets, sintered at 1150 °C for 48h, XRD collected in 2θ angle from 2° to 90° with a PANalytical CubiX fast diffractometer, using CuKα1 radiation (λ=1.5406 Å) and an X'Celerator detector in Bragg-Brentano geometry, GSAS II software used for refinement..
BSrPCF	$Ba_{0.5}Sr_{0.5}PrCo_{1.5}Fe_{0.5}O_{6-\delta}$	0.02	na	□	cubic $Pm\bar{3}m$	a = 3.844(1) Å	Rwp = 21.4%<br>Chi^2 = 1.7	Powder from crushed pellets, sintered at 1100 °C for 48h, XRD collected in 2θ angle from 15° to 85° with a Phillips X'Pert Pro diffractometer with Cu Kα radiation, proportional counter, and Bragg-Brentano geometry, GSAS II software used for refinement
BPNC55	$BaPr_{0.5}Nd_{0.5}Co_2O_{6-\delta}$	0.01	na	□	orthorhombic $Pmmm$	a = 3.91508(5) Å<br>b = 7.8121(1) Å<br>c = 7.6235(1) Å	Rwp = 3.0%<br>Chi^2 = 1.8	Powder from crushed pellets, sintered at 1150 °C for 48h, XRD collected in 2θ angle from 15° to 100° with a PANalytical CubiX fast diffractometer, using CuKα1 radiation (λ=1.5406 Å) and an X'Celerator detector in Bragg-Brentano geometry, GSAS II software used for refinement.
BSC	$BaSmCo_2O_{6-\delta}$	0.01	na	□	tetragonal $P4/mmm$ 25 wt %.<br>orthorhombic $Pmmm$ 75 wt %	a = 3.8962(1) Å<br>c = 7.5757(4) Å<br>-------------------<br>a = 3.88795(7) Å<br>b = 7.8375(2) Å<br>c = 7.5603(1) Å	Rwp = 7.8%	Powder from crushed pellets, sintered at 1150 °C for 48h, SR-PXD data used for refinement, ref: https://doi.org/10.1016/j.actamat.2020.08.018
BLSmC55	$BaLa_{0.5}Sm_{0.5}Co_2O_{6-\delta}$	0.01	na	□	tetragonal $P4/mmm$	a = 3.90642(5) Å<br>c = 7.6477(1) Å	Rwp = 2.5%<br>Chi^2 = 1.4	Powder from crushed pellets, sintered at 1150 °C for 48h, XRD collected in 2θ angle from 15° to 100° with a PANalytical CubiX fast diffractometer, using CuKα1 radiation (λ=1.5406 Å) and an X'Celerator detector in Bragg-Brentano geometry, GSAS II software used for refinement. Cell code 153495-ICSD
BTC	$BaTbCo_2O_{6-\delta}$	0.01	na	▽	orthorhombic $Pmmm$	a = 3.8667(2) Å<br>b = 7.81256(5) Å<br>c = 7.51259(5) Å	Rwp(SR-PXD) = 5.0%	Powder from crushed pellets, sintered at 1150 °C for 48h, combined PND and SR-PXD refinement, ref: https://doi.org/10.1016/j.actamat.2020.08.018

							Rwp(PND) = 5.5% Rwp(total) = 5.0%	
BNGC55	$BaNd_{0.5}Gd_{0.5}Co_2O_{6-\delta}$	0.01	na	□	orthorhombic *Pmmm*	a = 3.87801(5) Å b = 7.8182 (2) Å c = 7.5595(1) Å	Rwp = 2.3% Chi² = 1.8	Powder from crushed pellets, sintered at 1150 °C for 48h, XRD collected in 2θ angle from 15° to 100° with a PANalytical CubiX fast diffractometer, using CuKα1 radiation (λ=1.5406 Å) and an X'Celerator detector in Bragg-Brentano geometry. GSAS II software used for refinement.
BNGC28	$BaNd_{0.2}Gd_{0.8}Co_2O_{6-\delta}$	0.01	na	⊕	orthorhombic *Pmmm*	a = 3.90924(7) Å b = 7.8075(1) Å c = 7.6296(1) Å	Rwp = 2.5% Chi² = 1.7	Powder from crushed pellets, sintered at 1150 °C for 48h, XRD collected in 2θ angle from 15° to 100° with a PANalytical CubiX fast diffractometer, using CuKα1 radiation (λ=1.5406 Å) and an X'Celerator detector in Bragg-Brentano geometry. GSAS II software used for refinement.
BLNC28	$BaLa_{0.2}Nd_{0.8}Co_2O_{6-\delta}$	0	na	□	orthorhombic *Pmmm*	a = 3.9092(1) Å b = 7.8075(1) Å c = 7.6296(1) Å	Rwp = 2.5% Chi² = 1.7	Powder from crushed pellets, sintered at 1150 °C for 48h, , XRD collected in 2θ angle from 15° to 100° with a PANalytical CubiX fast diffractometer, using CuKα1 radiation (λ=1.5406 Å) and an X'Celerator detector in Bragg-Brentano geometry., GSAS II software used for refinement.
BNC	$BaNdCo_2O_{6-\delta}$	0	na	○	orthorhombic *Pmmm*	a = 3.9090(1) Å b = 7.8050(2) Å c = 7.6163(1) Å	Rwp(SR-PXD) = 6.9% Rwp(PND) = 4.5% Rwp(total) = 5.5%	Powder from crushed pellets, sintered at 1150 °C for 48h, combined PND and SR-PXD refinement, ref: https://doi.org/10.1016/j.actamat.2020.08.018
BLNC55	$BaLa_{0.5}Nd_{0.5}Co_2O_{6-\delta}$	0	na	□	orthorhombic *Pmmm*	a = 3.9046(1) Å b = 7.7892(2) Å c = 7.6745(2) Å	Rwp = 3.5% Chi² = 2.5	Powder from crushed pellets, sintered at 1150 °C for 48h, XRD collected in 2θ angle from 15° to 100° with a PANalytical CubiX fast diffractometer, using CuKα1 radiation (λ=1.5406 Å) and an X'Celerator detector in Bragg-Brentano geometry. GSAS II software used for refinement.
BGLLuC622	$BaGd_{0.6}La_{0.2}Lu_{0.2}Co_2O_{6-\delta}$	0	na	◆	tetragonal *P4/mmm*	a = 3.8891(1) Å c = 7.5685(1) Å	Rwp = 1.4% Chi² = 3.7	Powder from crushed pellets, sintered at 1150 °C for 48h, XRD collected in 2θ angle from 10° to 135° with a Phillips X'Pert Pro diffractometer with Cu Kα radiation, Pixcel 1D detector, and Bragg-Brentano geometry, GSAS II software used for refinement. Minor impurity peaks detected.

BGLLuC721	$BaGd_{0.7}La_{0.2}Lu_{0.1}Co_2O_{6-\delta}$	0	na	●	tetragonal<br>$P4/mmm$	a = 3.8917(1) Å<br>c = 7.5701(1) Å	Rwp = 1.7%<br>Chi^2 = 4.0	Powder from crushed pellets, sintered at 1150 °C for 48h, XRD collected in 2θ angle from 10° to 135° with a Phillips X'Pert Pro diffractometer with Cu Kα radiation, Pixcel 1D detector, and Bragg-Brentano geometry, GSAS II software used for refinement. Minor impurity peaks detected.
BPC	$BaPrCo_2O_{6-\delta}$	0	na	△	orthorhombic<br>$Pmmm$	a = 3.864(2) Å<br>b = 7.716(3) Å<br>c = 7.541(3) Å	Rwp(SR-PXD) = 7.9%<br>Rwp(PND) = 4.8%<br>Rwp(total) = 6.2%	Powder from crushed pellets, sintered at 1150 °C for 48h, combined PND and SR-PXD refinement, ref: https://doi.org/10.1016/j.actamat.2020.08.018
BNGC82	$BaNd_{0.8}Gd_{0.2}Co_2O_{6-\delta}$	0	na	□	tetragonal<br>$P4/mmm$	a = 3.8995(3) Å<br>c = 7.6000(7) Å	Rwp = 2.0%<br>Chi^2 = 4.3	Powder from crushed pellets, sintered at 1150 °C for 48h, XRD collected in 2θ angle from 10° to 135° with a Phillips X'Pert Pro diffractometer with Cu Kα radiation, Pixcel 1D detector, and Bragg-Brentano geometry, GSAS II software used for refinement. Minor impurity peaks detected.

Supplementary Figures

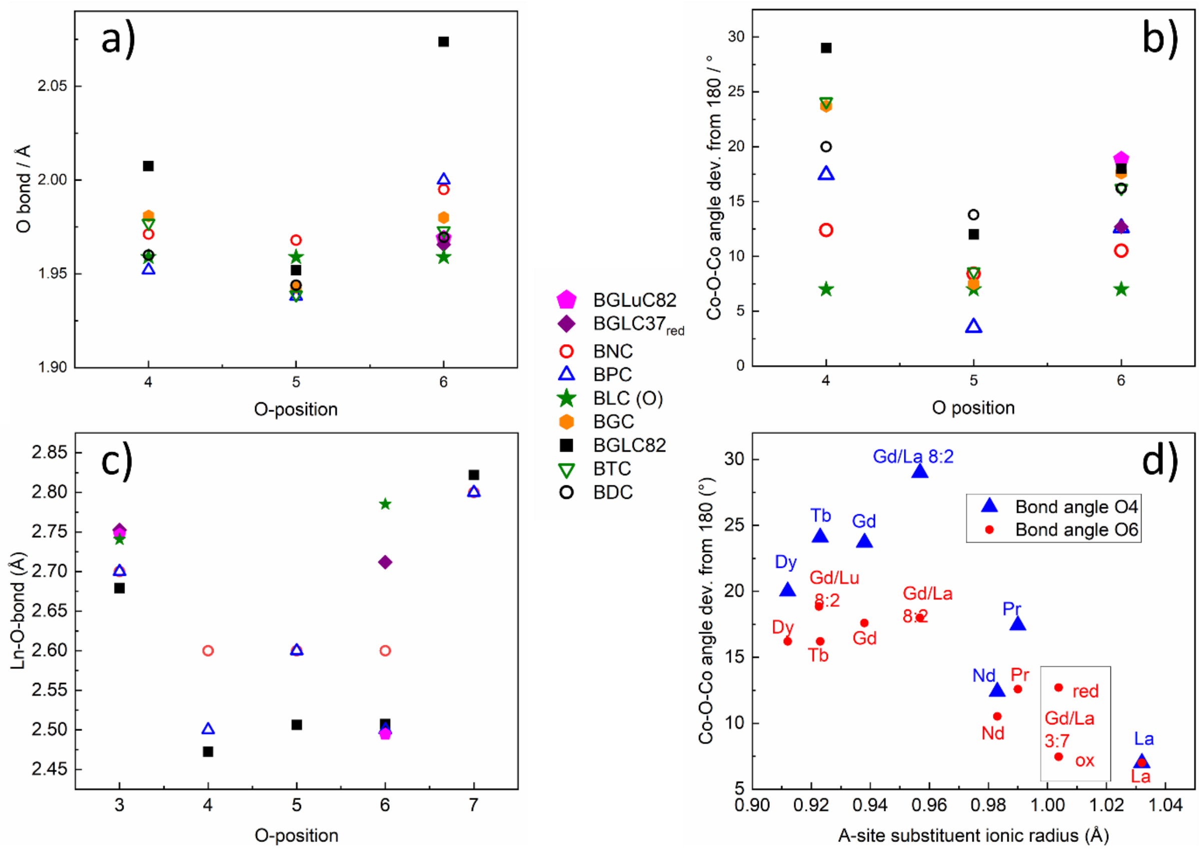

Supplementary Figure 1: Structural bond angles and lengths: Co-O bond lengths (a) and O-Co-O bond angle deviations from 180° (b) for O positions 4, 5 and 6 in a selection of closed and non-closed f-shell compositions. c) RE-O bond-lengths for O positions 3-7. d) O-Co-O bond angle deviations from 180° versus average Ln ionic radius. Source data are provided as a Source Data file.

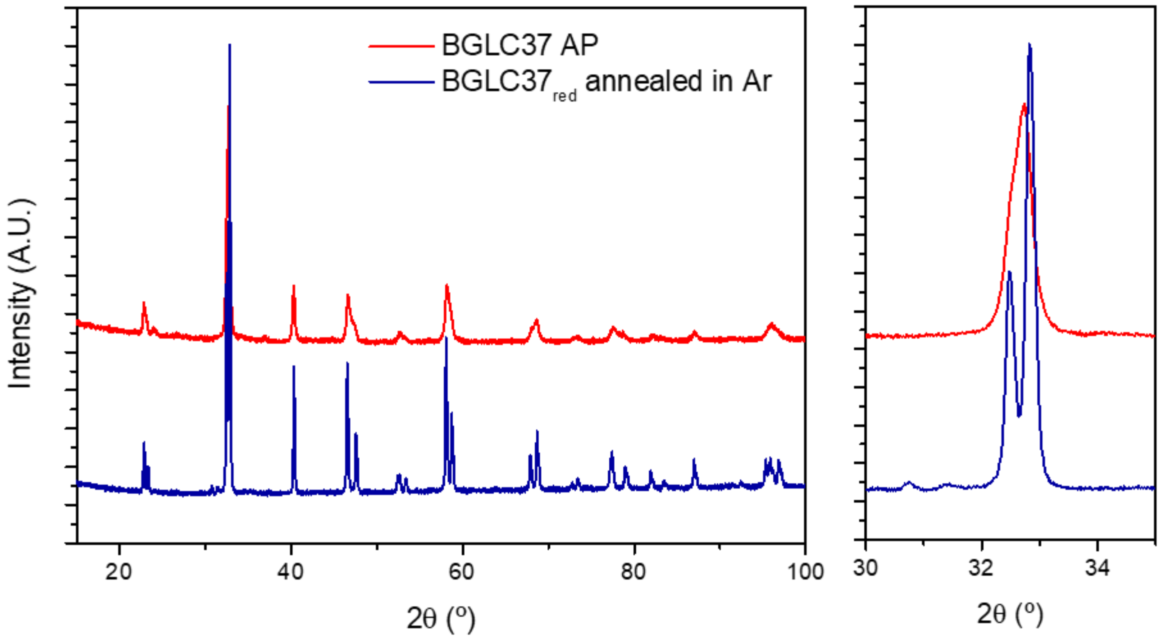

Supplementary Figure 2: XRD diffractograms of as-prepared BGLC37 as prepared (AP, Marion technologies, red) and BGLC37$_{red}$ after annealing in Ar for 10 h at 1000 °C and subsequently for 24 h at 350 °C in ambient air (blue).

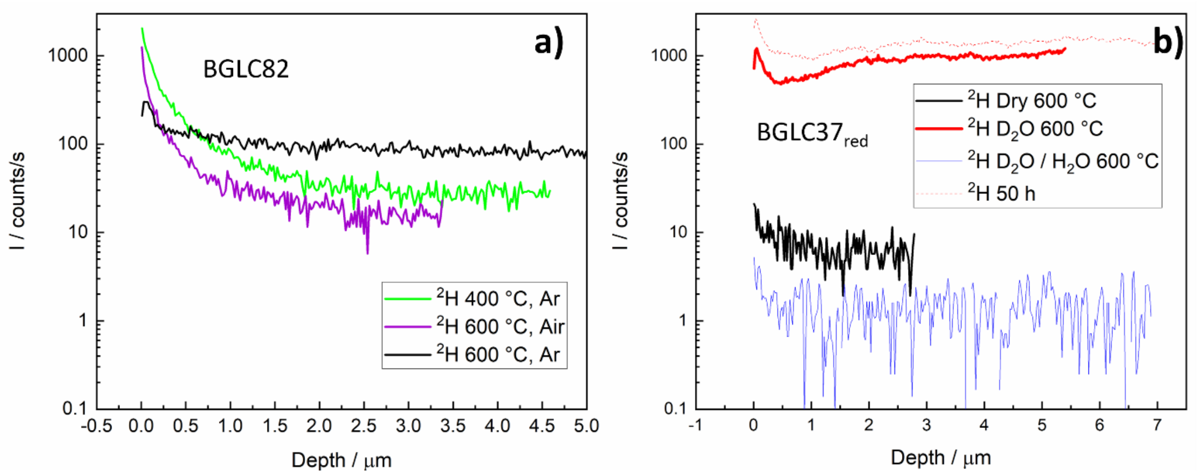

Supplementary Figure 3: SIMS concentration profiles of ^{2}H in BGLC82 (a) and BGLC37$_{red}$ (b). Black lines in b) represent dry state, and the blue line is after back-exchange from ^{2}H to ^{1}H.

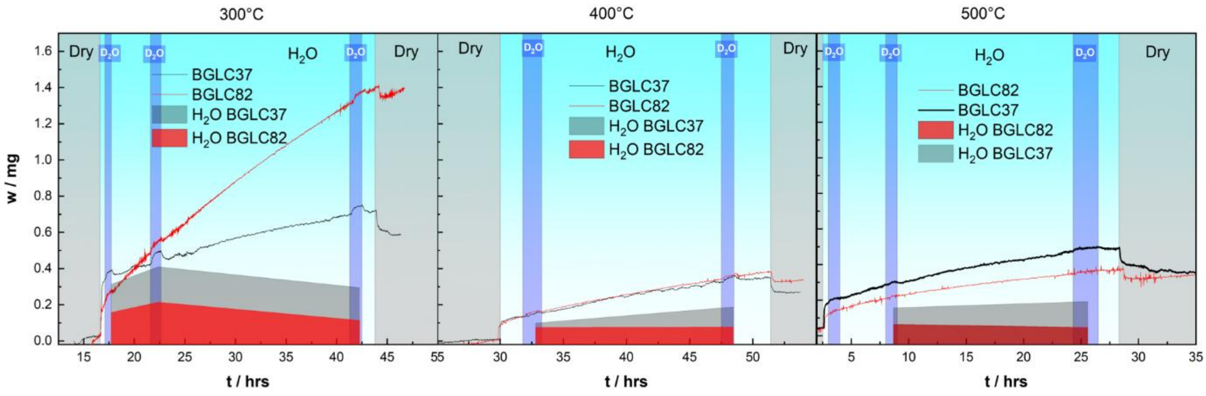

Supplementary Figure 4: TG curves and water uptake in BGLC82 and BGLC37 by changing from dry to humid air at 300-500°C.

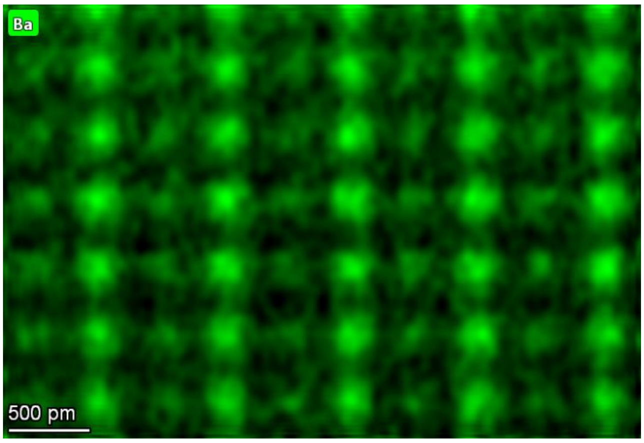

Supplementary Figure 5: HR-TEM image of ordering of Ba in BGLC37 in the dry state.

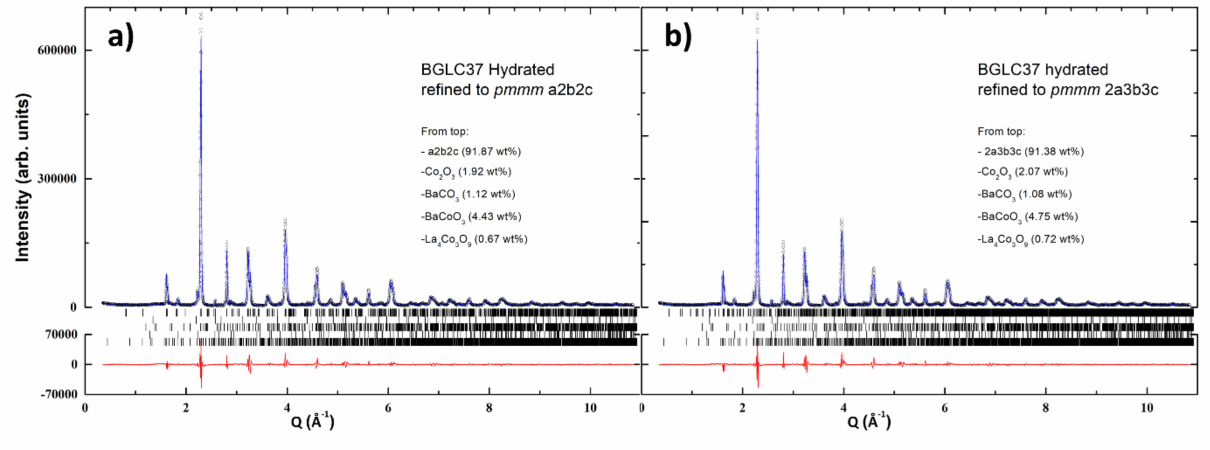

Supplementary Figure 6: Neutron diffraction: Hydrated BGLC37 refined to *Pmmm* a2b2c (a) and 2a3b3c (b). Minority phases are listed in the legends.

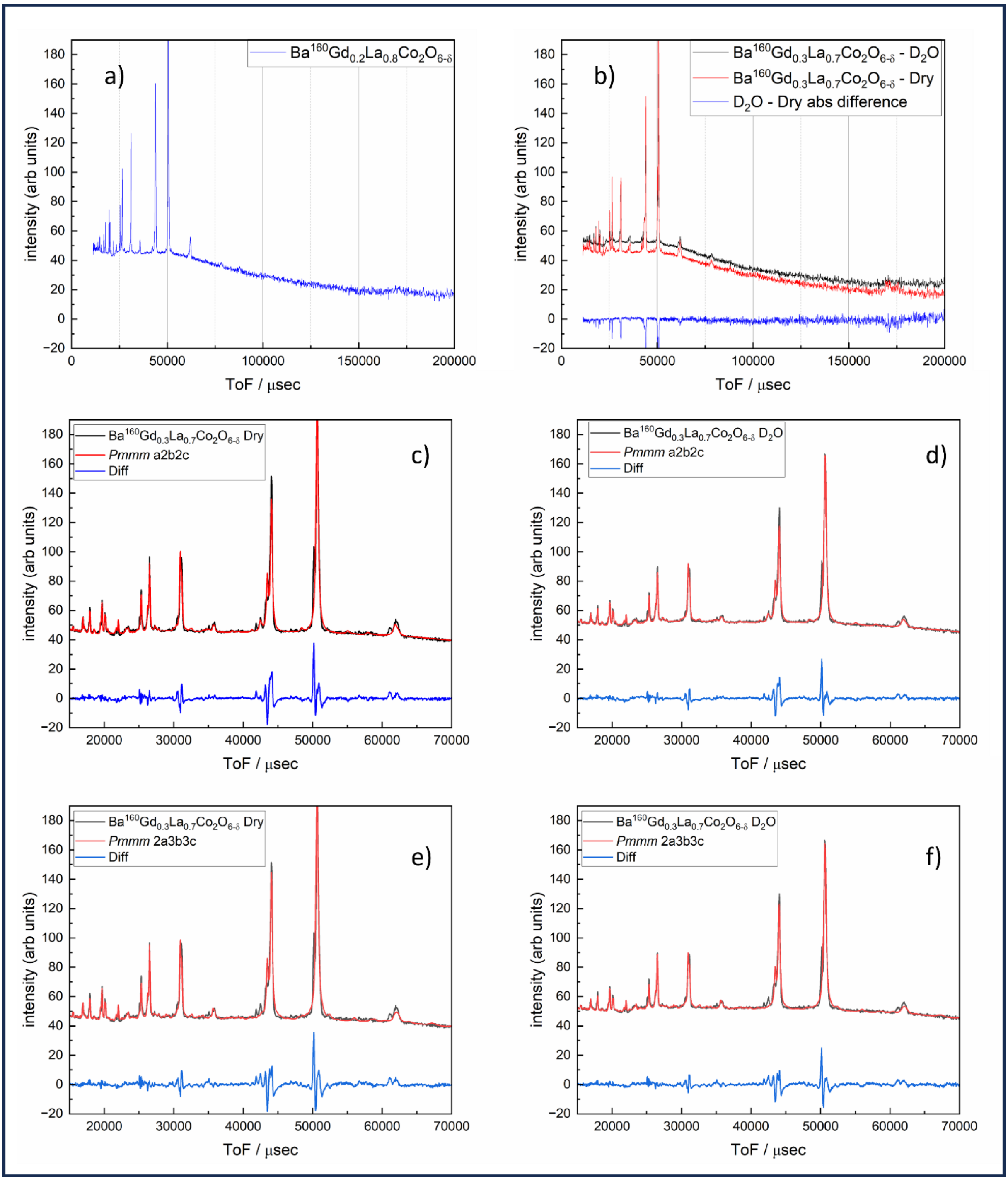

Supplementary Figure 7: ND diffractograms of ^{160}Gd-isotope enriched BGLC28 (a) and ^{160}Gd-isotope enriched BGLC37 (b) after treatments in D_2O (black) and dry (red) atmosphere. The absolute difference between the two datasets are given by the blue line. ^{160}Gd-isotope enriched BGLC37 in dry state refined to *Pmmm* a2b2c (c) and *Pmmm* 2a3b3c (e). ^{160}Gd-isotope enriched BGLC37 after treatments in D_2O refined to *Pmmm* a2b2c (d) and *Pmmm* 2a3b3c (f).

Supplementary Methods

Refinement parameters for neutron powder diffraction

Dry dataset, a2b2c:

Lattice parameters:
Space Group: *Pmmm*
a = 3.9037 Å
b = 7.8087 Å
c = 7.7066 Å

Fit parameters:
Rwp = 2.5595
GOF = 5.0711
R,bragg = 3.2039

Refined atomic parameters (errors: 0.001-0-002)

	x	y	z
RE1	0.5	0.2525	0.5
Ba1	0.5	0.2522	0
Co1	0	0.5	0.2362
Co2	0	0	0.2664
O1	0	0	0
O2	0	0.5	0
O3	0	0.5	0.5
O4	0.5	0	0.2814
O5	0.5	0.5	0.2420
O6	0	0.2624	0.2801
O7	0	0	0.5

Dry dataset, 2a3b3c:

Lattice parameters:
Space Group: *Pmmm*
a = 7.8075 Å
b = 11.5572 Å
c = 11.7136 Å

Fit parameters:
Rwp = 2.5274
GOF = 5.0392
R,bragg = 1.9751

Refined atomic parameters (errors: 0.001-0.005)

	x	y	z
RE1	0.5	0.1722	0.1655
RE2	0	0.1786	0.1747
RE3	0.5	0.5	0.1684
RE4	0	0.5	0.1788
RE5	0.5	0.1660	0.5
RE6	0	0.1444	0.5
RE7	0.5	0.5	0.5
RE8	0	0.5	0.5
Co1	0.2673	0.3532	0.3298
Co2	0.2480	0.3383	0
Co3	0.2545	0	0.3166
Co4	0.2451	0	0
O1	0.2538	0.3282	0.1621
O2	0.2539	0.1490	0.3307
O3	0.2449	0.3709	0.5
O4	0.2518	0.1640	0
O5	0.2625	0.5	0.3326
O6	0.2528	0	0.1600
O7	0.5	0.3307	0.3256
O8	0	0.2956	0.3531
O9	0.5	0	0.3267
O10	0	0	0.4271
O11	0.5	0.3404	0
O12	0	0.3891	0
O13	0.2757	0.5	0
O14	0.2793	0	0.5
O15	0.5	0	0
O16	0	0	0

Hydrated dataset, a2b2c:

Lattice parameters:
Space Group: *Pmmm*
a = 3.9026 Å
b = 7.8059 Å
c = 7.7035 Å

Fit parameters:
Rwp = 1.9040
GOF = 4.3635
R,bragg = 1.9412

Refined atomic positions **(errors: 0.001-0.003)**:

	x	y	z
RE1	0.5	0.2523	0.5
Ba1	0.5	0.2527	0
Co1	0	0.5	0.2353
Co2	0	0	0.2681
O1	0	0	0
O2	0	0.5	0
O3	0	0.5	0.5
O4	0.5	0	0.2815
O5	0.5	0.5	0.2418
O6	0	0.2642	0.2810
O7	0	0	0.5

Hydrated dataset, 2a3b3c:

Lattice parameters:
Space Group: *Pmmm*
a = 7.8062 Å
b = 11.5565 Å
c = 11.7097 Å

Fit parameters
Rwp = 1.8655
GOF = 4.2695
R,bragg = 1.1190

Refined atomic positions **(errors: 0.001-0.002)**:

	x	y	z
RE1	0.5	0.1742	0.1666
RE2	0	0.1712	0.1744
RE3	0.5	0.5	0.1679
RE4	0	0.5	0.1736
RE5	0.5	0.1676	0.5
RE6	0	0.1452	0.5
RE7	0.5	0.5	0.5
RE8	0	0.5	0.5
Co1	0.2735	0.3540	0.3188
Co2	0.2468	0.33402	0
Co3	0.2589	0	0.32523
Co4	0.2528	0	0
O1	0.2560	0.3261	0.16143
O2	0.2495	0.1478	0.33148
O3	0.2264	0.3775	0.5
O4	0.2550	0.1603	0
O5	0.2634	0.5	0.3313
O6	0.2538	0	0.1608
O7	0.5	0.3435	0.3259
O8	0	0.2991	0.3482
O9	0.5	0	0.3266
O10	0	0	0.4291
O11	0.5	0.3389	0
O12	0	0.3946	0
O13	0.2723	0.5	0
O14	0.2766	0	0.5
O15	0.5	0	0
O16	0	0	0

Supplementary Discussion

Hydration of a secondary phase

In recent literature,[1] hydration and phase purity of $BaGd_{1-x}La_xCo_2O_{6-\delta}$ were studied, and the authors suggested that the hydration seen for various compositions of BGLC stems from hydration of exsolved secondary phase $BaCo_{0.8}Gd_{0.2}O_3$ (BCG). We have therefore investigated the possibility of a contribution to the hydration from this secondary phase. By use of both SR-PXD and Powder Neutron Diffraction (PND), we have determined the phase fraction of BCG in all hydrating compositions, synthesized BCG and characterised its hydration to quantify partial contribution to the overall water uptake. As can be seen in Supplementary Figure 8, BCG shows a weight gain of 0.017% upon exposure to wet air at 300°C. Rietveld refinements of high-resolution SR-PXD data show that the phase fractions of BCG in the hydrating compositions range from zero (BGLC37) to 1.6 wt% ($BaGd_{0.8}Lu_{0.2}Co_2O_{6-\delta}$). BCG shows similar weight gain as the majority phases (Figure 2a) when shifting from dry to wet atmosphere at 300°C in air. Based on its phase fraction, hydration in BCG would have to be around 100 times higher to account

for the weight gain recorded in the main phases. This is clearly not the case. Furthermore, the weight gain in wet conditions for BLC cannot be rationalized by this secondary phase, since La is highly unlikely to substitute for Co on the B-site. We therefore respectfully disagree with the conclusion in the referred work[1] that hydration in BGLC is due to BCG only.

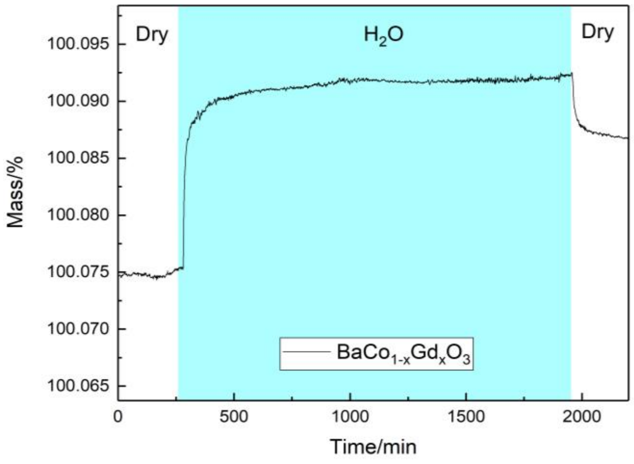

Supplementary Figure 8: Hydration of $BaCo_{0.8}Gd_{0.2}O_3$ at 300°C in air.

Supplementary Reference

[1] D. Malyshkin, A. Novikov, I. Ivanov, V. Sereda, D. Tsvetkov, A. Zuev, *Journal of Alloys and Compounds* **845** (2020) 156309.
